# Concomitancy of Type 1 Diabetes in Two Siblings: A Case Report and Review of Possible Viral Etiology

**DOI:** 10.7759/cureus.84296

**Published:** 2025-05-17

**Authors:** Ibrahim M Jely, Murouj A Almaghrabi, Elsayed M Elsalamony

**Affiliations:** 1 College of Medicine, Umm Al-Qura University, Makkah, SAU; 2 Preventive Medicine Department, Makkah Health Cluster, Makkah, SAU; 3 Department of Internal Medicine, Diabetes and Endocrinology Unit, Mansoura University, Mansoura, EGY

**Keywords:** concomitancy, coxsackievirus b3, diabetic ketoacidosis (dka), type 1 diabetes mellitus (t1d), viral infection

## Abstract

Type 1 diabetes mellitus (T1DM) is a chronic autoimmune condition with high prevalence in Saudi Arabia. Despite extensive research, the exact etiology remains unclear. Viral infections, particularly enteroviruses like coxsackievirus, have been proposed as potential triggers. In this case report, we describe two siblings who were diagnosed with T1DM a month apart, raising a clinical question regarding possible infectious triggers. We also included a comprehensive literature review of enteroviral contribution to the clinical onset of T1DM. A 17-year-old male, previously healthy, presented to the emergency department with diabetic ketoacidosis (DKA) following a two-day history of shortness of breath, epigastric pain, and fatigue. Laboratory investigations confirmed hyperglycemia, metabolic acidosis, and positive autoantibodies, confirming the diagnosis of T1DM. Remarkably, his 12-year-old younger brother was diagnosed with T1DM one month earlier, following a similar acute presentation. Both siblings denied recent viral symptoms, yet their close living conditions and shared use of objects may suggest the possibility of a common asymptomatic viral infection. The unique clinical presentation of the concomitant onset of T1DM in two siblings suggests a potential shared environmental or infectious trigger. Although both siblings denied a previous history of viral symptoms, the possibility of asymptomatic viral infection is still raised. This case emphasizes the need to explore causation relationships between enteroviruses and clinical T1DM. The findings emphasize the importance of heightened awareness and proactive research to identify and mitigate environmental contributors to T1DM onset. Identifying environmental triggers of T1DM will enable targeted prevention, early detection, and intervention strategies, ultimately reducing the disease burden and improving public health outcomes.

## Introduction

Type 1 diabetes mellitus (T1DM) is an autoimmune disease characterized by the immune-mediated destruction of insulin-producing pancreatic beta cells, resulting in hyperglycemia and lifelong insulin dependence [1]. Globally, T1DM affects millions of children and adolescents, and its burden continues to rise, particularly in regions with high genetic susceptibility and environmental risk factors.

According to the 8th edition of the Diabetes Atlas, Saudi Arabia ranks among the countries with the highest incidence of T1DM, with an estimated 35,000 affected children and adolescents and an incidence rate of 33.5 per 100,000 individuals. This places Saudi Arabia as the 8th most prevalent and 4th most incident country worldwide [2]. Despite this high burden, the precise etiology of T1DM remains unclear. While genetic predisposition plays a significant role - especially with specific human leukocyte antigen (HLA) haplotypes (e.g., DR3, DR4) - environmental factors, including viral infections, have been increasingly recognized as possible triggers for disease onset [1].

Among the suspected environmental factors, enteroviral infections, particularly coxsackievirus B (CVB), have received considerable attention due to their potential to induce autoimmune responses against pancreatic beta cells [3]. These viruses have been detected in the pancreatic tissues of individuals with T1DM and are hypothesized to initiate beta-cell destruction through molecular mimicry and immune activation [3].

In this case report, we describe two siblings diagnosed with T1DM within a month of each other. Both presented with acute symptoms and positive autoimmune markers for T1DM, but denied prior symptoms of viral illness. Notably, the siblings live in the same household and share personal belongings, raising the possibility of a shared subclinical or asymptomatic environmental exposure.

This case prompts reflection on the role of potential non-genetic triggers in familial clustering of T1DM, especially within a high-prevalence country like Saudi Arabia. We aim to contribute to the growing body of literature suggesting a link between enteroviral exposure and the rapid onset of T1DM, and we advocate for further research exploring preventive strategies.

## Case presentation

A 17-year-old male, previously healthy, presented to the emergency department (ED) of our hospital at the Security Forces Hospital, Makkah, Saudi Arabia, complaining of a 2-day history of shortness of breath (SOB), epigastric abdominal pain described as a burning sensation accompanied by symptoms of gastroesophageal reflux disease (GERD), nausea, vomiting of gastric and food contents, and generalized fatigue. The complaint had progressively worsened since it started. There are no known allergies, and the patient denied recent travel, contact with sick patients, recent infections, trauma, or exposure to toxins.

On examination, the patient appeared fatigued and dehydrated. Weight dynamics: height: 167 cm, weight: 55 kg, body surface area (BSA): 1.60, and body mass index (BMI): 19.72. His vital signs were as follows: temperature: 36.9°C, heart rate: 92 beats per minute, blood pressure: 112/74 mmHg, respiratory rate: 16 breaths per minute, and oxygen saturation: 99% on room air. Abdominal examination revealed mild tenderness in the epigastric region without guarding or rebound tenderness. Cardiovascular, respiratory, and neurological examinations were unremarkable.

Laboratory diagnostic investigations were performed and demonstrated in Table 1. Hence, it reveals elevated random blood sugar (RBS): 388 mg/dl, hemoglobin A1c: 12.6%, and the presence of protein, glucose, and ketones in the urine. The arterial blood gas (ABG) values indicate metabolic acidosis (PH: 7↓, HCO_3_: 14↓, PCO_2_: 14↓) with a high anion gap (27↑) (Reference range: PH: 7.35-7.45, HCO_3_: 22 - 26 mmHg, PCO_2_: 35 - 45).

**Table 1:** Hematological and biochemical parameters during diagnostic workup for both siblings Bold values indicate critical abnormalities. RBS and HbA1c confirm severe hyperglycemia and poor long-term glycemic control. pH, low HCO₃, and low PaCO₂ reflect high anion gap metabolic acidosis with respiratory compensation—hallmarks of DKA. Positive GAD autoantibodies confirm autoimmune etiology consistent with T1DM. Urine glucose (4+) and ketones (2+) support the presence of glycosuria and ketonuria typical of DKA.

Parameter	Sibling 1 (17-year-old)	Sibling 2 (12-year-old)	Reference Range(Units)
Random Blood Sugar (RBS)	388	375	70.2 – 140.5 (mg/dL)
Hemoglobin A1C (HbA1C)	12.60	11.2	4.8-5.9 (%)
GAD autoantibodies	Positive	Positive	Negative
Blood gases			
pH	7.00	7.23	7.35 - 7.45
PaCO_2_	14	17	35 – 45
HCO_3_	14	13.9	22 - 26 mmHg
Sodium	136.1	148	136-145 (mmol/L)
Potassium	3.47	3.7	3.5-5.1 (mmol/L)
Chloride	95.07	120	98-107 (mmol/L)
Lactate	1.05	2.5	0.5-2.2 (mmol/L)
Urine analysis			
pH	6.0	6.0	5-6
Protein	1+	1+	Negative
Glucose	4+	4+	Negative
Ketones	2+	5.3	Negative
Blood	Negative	Negative	Negative
Bilirubin	Negative	Negative	Negative

Based on these findings, the patient was diagnosed with T1DM and diabetic ketoacidosis (DKA). He was admitted to our Department of Internal Medicine for management with intravenous fluids, insulin therapy, and electrolyte replacement.

Notably, the patient’s 12-year-old younger brother was diagnosed with T1DM one month earlier, following a similar acute presentation of a 1-week history of polyurea, polydipsia, and abdominal pain. Physical examination on admission revealed normal vitals and weight dynamics of height: 154 cm, weight: 54 kg, BSA: 1.52, and BMI: 22.77. Laboratory investigations revealed elevated random blood sugar, HbA1c, and positive glutamic acid decarboxylase (GAD) autoantibodies support the diagnoses of T1DM. In addition, abnormal blood gas values and ketones align with the presence of DKA (Table 1). Prior to his diagnosis with T1DM, the patient denied any history of fever, rashes, or viral infection symptoms. Nevertheless, both brothers share the same room and share the same objects. The parents also denied a family history of T1DM and consanguineous marriage.

Following discharge, both siblings were enrolled in routine outpatient follow-up clinics. They have remained clinically stable on a basal-bolus insulin regimen with regular glucose monitoring and nutritional support. No complications or hospital readmissions were reported during follow-up, and both patients have demonstrated good adherence to their diabetes management plans.

## Discussion

In our case, we observed the concomitant onset of T1DM in two siblings diagnosed within a month of each other, which raises the question of potential shared etiological factors. While genetic predisposition is a known contributor to T1DM, the near-concurrent diagnoses suggest an external trigger, such as a viral infection, which may be involved. Despite the absence of significant viral symptoms in either brother, asymptomatic viral infections such as coxsackievirus cannot be excluded, as more than 90% of patients with coxsackievirus do not exhibit any symptoms [4].

In the realm of T1DM, identifying the environmental etiologies was constantly a major challenge [5]. Reviewing the literature, a previous systematic review with meta-analysis of observational studies conducted at the University of New South Wales, Sydney, Australia found an association between type 1 diabetes and enterovirus infection, with more than nine times the risk of infection in cases of diabetes and three times the risk in children with autoimmunity, this analysis included 24 papers and two abstracts, with a total of 4,448 participants [6]. Another review study, supported by prospective and pancreas histopathology studies, explored the link between coxsackievirus B (CVB) infection and the development of T1DM and reported that CVB infection is associated with pancreatic islet autoimmunity and clinical T1DM, by the following suggested mechanisms: i) The direct pathogenic effects of beta-cell infection are caused by CVB, which could indicate ineffective immune responses that are unable to effectively eradicate the pathogen. ii) The indirect pathogenic effects of beta-cell infection caused by antiviral immune responses include, but are not limited to, cytotoxic CD8+ T-cell-mediated beta-cell lysis. And lastly, iii) The imitation of epitopes, as homologous CVB and beta-cell epitopes elicit T-cell cross-reactivity, the natural antiviral immune response may transform into a pathogenic autoimmune response against beta cells [7]. Nevertheless, there is substantial evidence linking CVB infections to islet autoimmunity, but a causative relationship is still lacking [8].

Over the past decade, significant progress has been made in understanding the potential viral etiology of T1DM. Hence, a recent study has provided more nuanced insights, indicating that while certain viral infections may be associated with T1D development, they likely interact with a complex interplay of genetic and environmental factors. Notably, a 2021 meta-analysis found an association between enterovirus infection and increased T1D risk, yet causation could not be conclusively established [9]. Additionally, studies have explored the role of viral infections in modulating gut microbiota, which may influence autoimmune responses related to T1D [10]. Interestingly, recent studies also highlighted the potential link between other enteroviruses like COVID-19 and T1DM, as viruses may trigger autoimmune responses by activating autoreactive immune cells or initiating inflammation in genetically predisposed individuals [11].

In reviewing the literature, there is a notable paucity of studies examining the simultaneous onset of T1DM among individuals sharing the same living environment without genetic ties. Existing research predominantly focuses on familial clustering and genetic predisposition, with limited exploration of environmental factors influencing T1DM development among non-related cohabitants. This gap underscores the necessity for further investigation into shared environmental exposures and lifestyle factors that may contribute to the concurrent emergence of T1DM in such populations.

Other well-known etiologies of T1DM include genetic factors; hence, the HLA region on chromosome 6p21 accounts for approximately 50% of the familial aggregation of T1D, particularly through specific HLA haplotypes such as HLA-DR3 and HLA-DR4, which are associated with increased risk. This highlights the significant role of genetic predisposition in the familial clustering of T1DM. Siblings with the high-risk DR3/DR4-DQ8 genotype who shared both haplotypes with their probands have about an 85% risk of T1DM by the age of 15 years. This high genetic risk might be relevant to our case, as both siblings developed T1DM within a short time of each other [12]. Furthermore, autoimmunity is another etiological factor. There are various autoantigens, and the corresponding autoantibodies are involved in the autoimmune destruction of pancreatic beta cells. This is important in T1DM pathogenesis, e.g., islet cell antibodies (ICA): Detected in many patients with T1DM, ICAs are among the earliest markers of autoimmune activity against beta cells. Insulin autoantibodies (IAA): Found in individuals with T1DM even before they receive insulin therapy. Their presence, especially in non-diabetic relatives, indicates a high risk of developing the disease. Glutamic acid decarboxylase (GAD65) Autoantibodies: GAD65 is a significant autoantigen in T1DM. Autoantibodies against GAD65 have been linked to the destruction of beta cells and are often present in the early stages of T1DM. The role of autoimmunity is further underscored by the presence of multiple autoantibodies, indicating that an aberrant immune response targeted the patient’s pancreatic beta cells [13].

The current study has demonstrated a unique presentation of concomitant onset of T1DM in two siblings, which raises further questions about the potential shared etiological factors and suggests future studies to address the causation relation between viral infections such as CVB and clinical T1DM. Nevertheless, our study has several limitations to disclose. First, the limited nature of the study design eliminates the ability to demonstrate an association between risk factors and the disease. Second, the absence of viral serology testing for enteroviruses. Although both siblings denied a history of viral illness, asymptomatic infection remains a plausible hypothesis, especially given the high rate of subclinical presentations in coxsackievirus infections. Without confirmatory serological data, the proposed infectious trigger cannot be definitively established, which limits the strength of causal inference in our study.

## Conclusions

This case report documents the concomitant diagnosis of T1DM in two siblings, aged 17 and 12 years, within a month of each other, both presenting with acute symptoms and confirmed hyperglycemia, DKA, and positive autoantibodies. These findings suggest a potential shared environmental or infectious trigger, possibly linked to enteroviruses like coxsackievirus. Although both siblings denied a previous history of viral symptoms, the possibility of asymptomatic viral infection is still raised, in addition to the close living conditions of the siblings. This case emphasizes the need to explore causation relationships between enteroviruses and clinical T1DM.
